# The Role of Voids in the Cracking of Single-Crystalline Composites with Quasicrystal Phase Fraction

**DOI:** 10.3390/ma18194506

**Published:** 2025-09-28

**Authors:** Jacek Krawczyk

**Affiliations:** Institute of Materials Engineering, Faculty of Science and Technology, University of Silesia in Katowice, 1a 75 Pułku Piechoty St., 41-500 Chorzów, Poland; jacek.krawczyk@us.edu.pl

**Keywords:** quasicrystals, composites, single-crystal, cracking, defects

## Abstract

The novel fibrous composites of Al_61_Cu_27_Fe_12_ alloy with a single-crystalline matrix and quasi-crystal phase fraction obtained in situ by directional solidification by the Bridgman method were studied to characterize the voids and their role in composites cracking. The voids were analyzed using light-optical and scanning electron microscopy to study their nature before and after uniaxial tensile tests. Tension tests were performed on plate-like samples up to rupture. The tensile fracture surfaces were also observed and analyzed. The single-crystallinity and crystalographic parameters of composites were studied using the X-ray Laue diffraction method. It was stated that such new type of composite is characterized by a relatively high void content with a ratio of approximately 2.6%. The composite’s cracking is initiated at voids and progress through the voids and stair steps in the matrix and the reinforcing fibers.

## 1. Introduction

The multiphase metallic composites are currently used as one of the main types of materials that can meet the high demands of the operating environment in aviation, automotive, space industries, and many others. The construction elements of jet engines, gas turbines, and automobile engines, especially in combustion chambers, are exposed to, inter alia, mechanical stress, high temperatures, and corrosion. It requires high mechanical strength, thermal, and corrosion resistance of the material they produce from, or thermal barrier coatings application with the possibility of fast, directional heat dissipation in the required way [1,2,3,4,5,6,7,8]. In such applications, metal matrix composites with metallic directional, continuous reinforcement are a good solution. The reinforcement not only strengthens the material but also facilitates directional heat dissipation [9,10,11]. It must contain a phase that has good thermal and strength properties.

The production method is very important for the properties of fiber-reinforced composites. A group of ex situ methods, including, for example, powder metallurgy, is the most frequently used for obtaining the composites with directional oriented fibrous reinforcement [12,13,14,15]. They are often used due to economic aspects, whilst maintaining appropriate material quality. However, the number of defects, especially the voids and weak bond between the matrix and reinforcement, reduces their quality and applicability [16,17,18,19]. These limitations can be minimized using in situ methods, especially directional solidification methods like the Bridgman technique. The Bridgman technique allows for the production of single-crystals, single-crystalline multiphase alloys, and composites containing a single-crystal matrix [20,21,22].

Defects created during composite production may be classified by their location into matrix defects, fiber defects, and interface defects. Fiber defects include fiber waviness, misalignment, and discontinuity. Interface defects include fiber/matrix debonding and delamination. Matrix defects include, e.g., grain/subgrain boundaries and spaces unfilled with atoms, i.e., voids. Voids are among the most notable defects due to their major effect on various composite properties and mechanisms leading to failure [17,23,24]. The tendency to form voids in composites is determined by the relationships between the properties of the components and the conditions and methods of their production. Generally, voids may arise from three main causes: gas entrapment during mixing and melting raw elements, hydrogen generation, and casting shrinkage during solidification [25]. When obtaining metallic composites by directional solidification, voids are created rarely, but if they are, it is mainly due to gas dissolution in the melt and the shrinkage of casting during solidification [16,26].

The literature reports on composites based on Al-Cu-Fe alloys containing quasicrystalline phases, which are obtained ex situ via hot press sintering, spark plasma sintering, or other powder metallurgy methods. Quasicrystal phases possess properties similar to ceramics: good stability at high temperatures, a low coefficient of sliding friction, and high hardness. However, their thermal conductivity is lower than that of metals and can be brittle. Difficulty with this composite type includes the relatively weak reinforcement–matrix bond and the non-continuous reinforcement [12,13,27,28,29,30,31]. These problems can be eliminated by a new type of composite obtained in situ via directional crystallization methods, where the matrix and the reinforcement in the form of oriented fibers are formed during a single solidification process. Additionally, to strengthen the bond between the matrix and the reinforcement, a quasicrystalline phase can be created between them [21,22,32]. Unfortunately, such a novel composite has not been widely studied and described. The only published studies concern preliminary phase analysis, and the stability of such composites was presented in [21]. No detailed studies have been presented to date to determine the mechanical properties, particularly potential sources of reduction in mechanical properties. It is known that some of such composites may be unstable or easily cracked [33,34,35]. In the case of directionally solidified composites, the reason may be defects formed during the process, e.g., voids [32,36,37,38]. No research is available on void creation in this new composite type and its influence on cracking.

Considering the above and lack of information about the character of voids and the reasons for cracking in composites of a single-crystalline phase matrix and reinforcement with a quasicrystalline phase fraction, the study aimed to characterize voids and determine their role in cracking a novel composite based on an Al_61_Cu_27_Fe_12_ alloy produced by the Bridgman method.

## 2. Materials and Methods

The composite production process was divided into two separate stages. In the first stage, raw chemical elements of a total mass of 40 g, in quantities determined based on the chosen chemical composition of Al_61_Cu_27_Fe_12_ (at.%), were placed in an alumina crucible and then melted and homogenized in the Garricast induction furnace of Mullard Intertherm, Blackburn, UK. The melting process was carried out in a protective atmosphere of high-purity helium (5N) under atmospheric pressure. The batch was gradually heated to approximately 1600 °C and then homogenized at this temperature for approximately 10 min. By cooling the alloy in the furnace, the ingots with a diameter of 16 mm and a height of 80 mm were obtained, resulting from the first production stage. The ingots were batch material for the second stage of composite production. The conical alumina crucible with the batch was placed in a growth chamber of the BCG-256 vertical induction heating furnace of Radyne Metals Research, Milwaukee, WI, USA, and melted in an inert atmosphere of He (5N) under atmospheric pressure. The maximum melt temperature measured on the top was approximately 1450 °C. After reaching the required temperature, the crucible with the melt was withdrawn from the high-temperature zone at a rate of 0.07 mm/min. producing composites. For further research, five conical ingots with a diameter of 16 mm and a height of approximately 75 mm were produced. Obtained ingots were cut into slices perpendicular and parallel to the withdrawal direction using electrical discharge machining. Such slices will hereinafter be referred to as parallel slices and perpendicular slices, respectively.

The middle fragments of the ingots were powdered in an agate mortar and were subjected to X-ray diffraction (XRD) phase analysis. The X-ray diffraction patterns were collected using the X’Pert Philips diffractometer of Philips Analytical, Alamelo, The Nederlands, equipped with a Cu X-ray tube and a curved graphite monochromator on the diffracted beam.

The single-crystallinity and crystalographic parameters of composites were studied using the X-ray Laue diffraction method. The Laue patterns were recorded on X-ray Agfa film using the X-ray diffractometer Laue system provided by TuR corporation (TuR GmbH, Hohen Neuendorf, Germany) from the analyzed sections. The white X-ray incident beam of Co radiation was used.

Microstructure observations were carried out by the Alphaphot 2 optical-light microscope (OLM) of Nikon Corporation, Tokyo, Japan, and the JMS-6480 scanning electron microscope (SEM) of JEOL Ltd., Akishima, Tokyo. The SEM microscope, equipped with an energy dispersive X-ray spectroscope (EDXS), was used for chemical microanalysis. Backscattered electrons (BSE) were applied to produce images with contrast that carries information about the atomic number differences in distinct phases. The secondary electron SE mode was used in the voids SEM observations because secondary electrons are very useful for inspecting the topography of the sample’s surface, including the voids. The geometrical parameters of voids arrangement and void ratio of the obtained composites were measured and calculated using ImageJ v.1.53, an image analysis software, based on OLM microstructure images.

Samples for microscopic observation were prepared by making metallographic microsections. The surfaces were ground using abrasive paper with grit sizes ranging from 300 to 600, 1000, 1500, and 2000, and then polished on felts using suspensions with abrasive sizes of 9 µm, 6 µm, 3 µm, and 1 µm. To reduce the possibility of identifying material defects created by mechanical polishing of a metallographic cross-section as defects created during the tensile test, chemical etching was used to reduce surface stresses and surface defects, as well as to make voids more visible. A difference in the etch rate between different areas can create a greater topographical contrast, making the voids more available for the electron beam, finally more visible and easier to distinguish from the surrounding phase. Since the β-phase had the largest volume fraction, an etchant reacting with this phase was chosen (40% Hydrofluoric acid (HF) for 15 s).

Simple uniaxial tension tests were performed on plate-like specimens (10 mm × 40 mm × 1.5 mm, gauge section: 7 mm × 20 mm × 1 mm) prepared from ten parallel slices to arrange the withdrawal axis parallel to the tensile axis. The special non-serial tensile testing machine constructed in the University of Silesia in Katowice under Polish Grant KBN No. 7T08A02615 was used for tensile tests. The machine was equipped with attachments for X-ray diffraction topography study and tests at elevated temperatures. The tests were carried out in static conditions at room temperature under an air atmosphere using a crosshead speed allowing a stress rate of 3 MPa/s.

## 3. Results and Discussion

**Figure 1:**
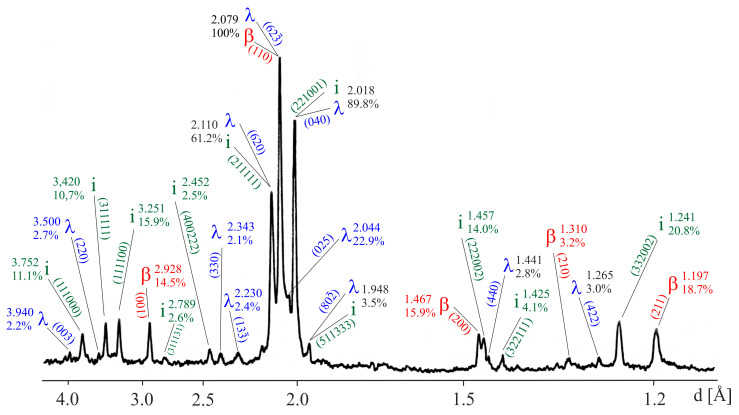
Typical powder XRD pattern of the obtained composites.

The experimental patterns (Figure 1) were compared to reference patterns found in the database of ICDD’s (International Centre for Diffraction Data) Powder Diffraction File (PDF^®^). The analysis of the XRD pattern allows the identification of three phases contained in the composite. The most intense peaks indicated the crystalline cubic β-phase (Al(Cu,Fe)). The peaks of crystalline monoclinic λ-phase (Al_13_Fe_4_) and quasicrystalline icosahedral i-phase (Al_6_Cu_2_Fe) were also identified. The obtained diffraction patterns and the position of diffraction peaks for individual phases are similar to those presented in [39,40] for Al-Cu-Fe alloys; still, they were obtained by different methods.

**Figure 2:**
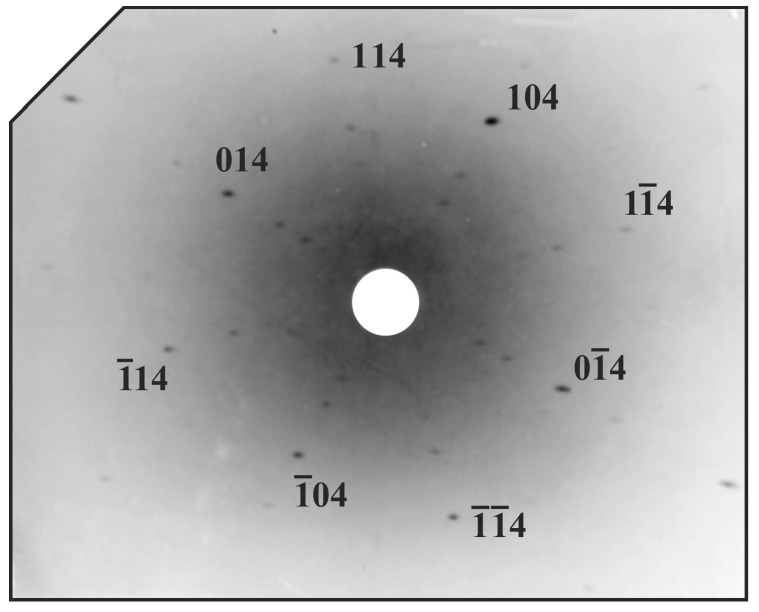
Typical Laue diffraction pattern, [001] direction.

The Laue diffraction patterns were collected from the metallographic sections of parallel and perpendicular slices. The example Laue pattern obtained from parallel slices along the [001] direction is presented in Figure 2. The pattern shows regular distribution of Laue spots, corresponding to the symmetry of the cubic β-phase. It indicates the diffraction beam was recorded mainly from a composite matrix formed by a dominant β-phase. Although some spots are slightly stretched or blurred, a symmetrical pattern with intense spots indicates that the β-phase is single-crystalline. These aberrations in spot quality may be related firstly to the broad energy distribution of the white beam X-ray source. This results in several overlapping diffraction harmonics and weaker individual reflections. Others, if some spots appear to be weak due to the background intensity related to the intensity of the primary X-ray beam passing through the center of the X-ray film (reflection geometry was used), and the scattering of the diffracted beam, most likely due to defects such as voids (creating a stress field), etc. The second reason may be a quasicrystalline aperiodic phase appearance, which is present in a smaller amount but is also covered by the incident X-ray beam.

**Figure 3:**
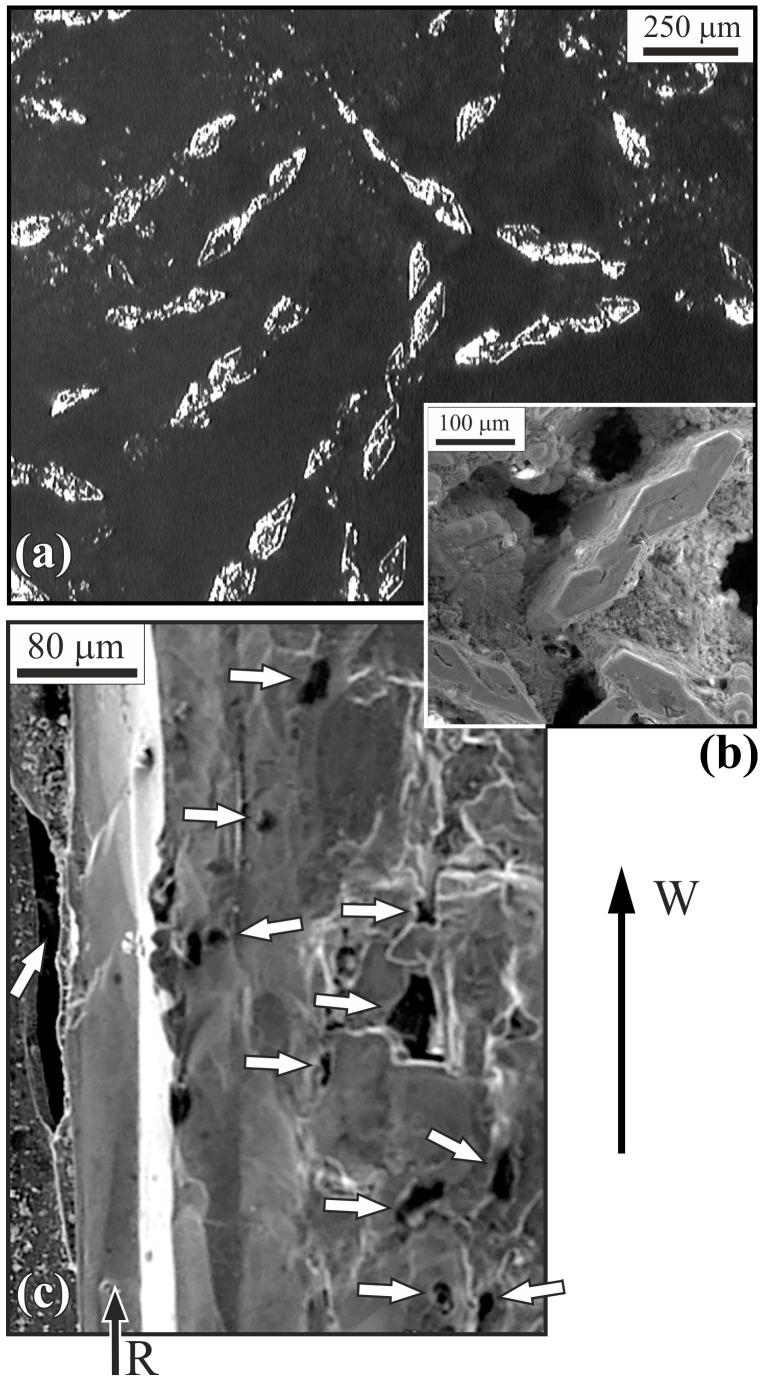
Micrographs of a typical microstructure of (**a**) perpendicular slices microsection, (OLM); (**b**) selected fragment of perpendicular slices microsection, (SEM); (**c**) parallel slices microsection, (SEM). R—reinforcement fiber, W—withdrawal direction, arrows indicate voids.

Microstructure observations were carried out using OLM and SEM on the metallographic sections prepared perpendicular and parallel to the withdrawal direction on both types of slices. Observed morphology was similar to Al-Cu-Fe composites obtained by directional crystallization methods with similar or the same withdrawal rates and described, for example, in [21]. The typical microstructure of perpendicular slices is presented in Figure 3a. The composites consisted of a matrix visible as a dark area and a reinforcement in the form of fibers with rhomboidal cross-sections visible as bright rhombi. The perpendicular slices were used to analyze reinforcement distribution, and parallel slices were used to analyze the continuity of fibers. The fibers were most often arranged in straight chains and were continuous along the entire length of the slices. A closer look at the matrix, for example, in the proximity of rhombs (Figure 3b), allows one to identify the empty spaces in the material—voids. Observation of parallel metallographic sections shows that the reinforcing fibers R (Figure 3c) had a columnar form and were arranged almost parallel to the withdrawal direction W. Analysis of the revealed microstructure of parallel sections confirms the existence of numerous voids in the matrix of the composites, as indicated by the arrows in the example micrograph presented in Figure 3c.

**Figure 4:**
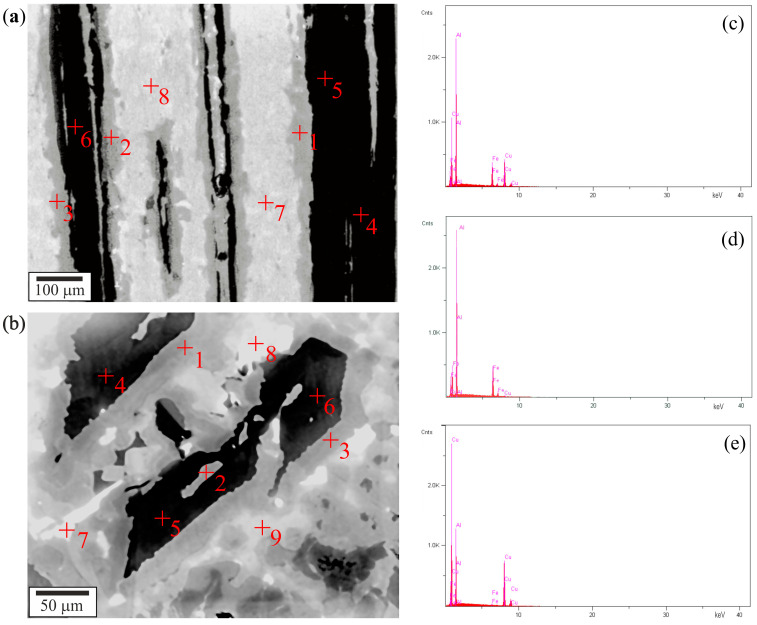
Micrographs of a typical microstructure of (**a**) parallel slices microsection, (SEM-BSE); (**b**) perpendicular slices microsection, (SEM-BSE) with marked points 1–9 of microanalysis; EDXS spectra obtained for (**c**) point 1 in figure (**a**,**d**) point 5 in figure (**a**,**e**) point 7 in figure (**a**).

Chemical composition analysis was implemented at selected points in the micrographs of the microstructure to identify individual areas in the micrographs and compare them to the component phases of the composite previously identified by XRD. The perpendicular slice microsection area, which was previously used to identify the phase composition, was used for chemical analysis [21]. The arrangement of component phases in the composite was determined based on measurements of the chemical composition at points located in the separated areas visible on the microstructure. Figure 4 shows the example of SEM (BSE) images with chemical analysis points, performed on perpendicular slices microsection (Figure 4a) and parallel slices microsection (Figure 4b). Figure 4c–e present examples of EDXS spectra, obtained for points 1, 5, and 7 marked in Figure 4a.

The concentration of elements in the analyzed points is presented in Table 1 (for Figure 4a) and Table 2 (for Figure 4b). Based on each phase’s measured elements ratio and stoichiometric formula, the phases visible on the SEM images were identified. It was determined that the grey area, where lay points 1–3, represents the i-phase; the black area, where lay points 4–6, represents the λ-phase; and the bright area, where lay points 7–9, represents the β-phase.

**Figure 5:**
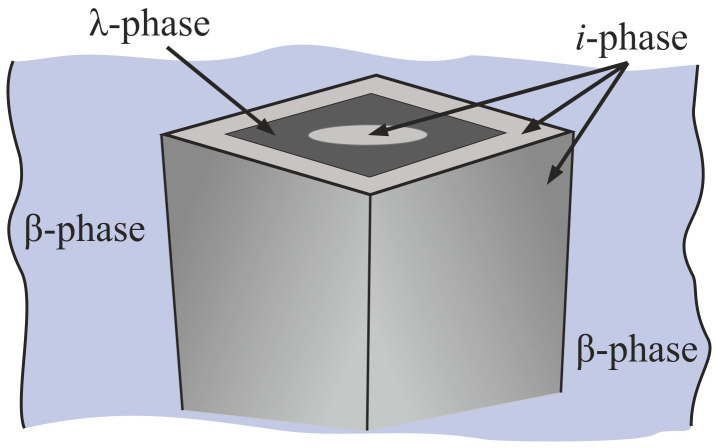
Schematic representation of phase arrangement in the composites.

Obtained microanalysis data allowed one to establish that the composite matrix was composed of a single-crystalline β-phase, and the fibrous reinforcement consisted of a crystalline λ-phase and a quasicrystalline i-phase, which was located in the core of the fibers and on their side surfaces, as shown in the schematic representation in Figure 5.

**Figure 6:**
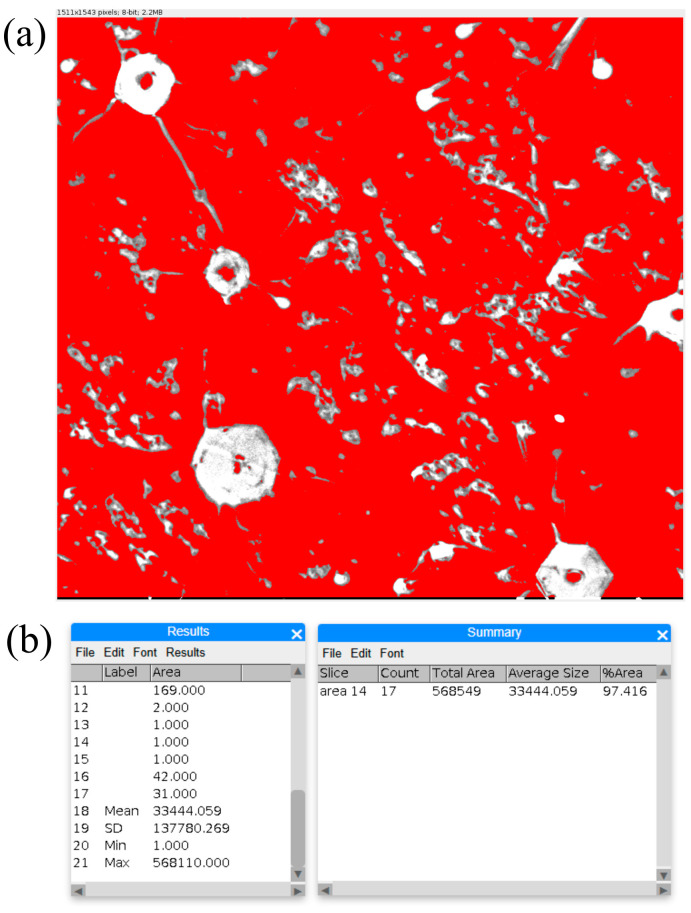
A typical (**a**) image of the software analysis processing (OLM), and (**b**) analysis data.

To quantify the void content in the composites, void ratio measurements were performed on OLM images obtained from five areas for each of the ten samples, using ImageJ image analysis software. A typical processed image during the analysis is presented in Figure 6a. Figure 6b presents raw data for voids size and ratio calculations. The areas presented in Figure 6a as bright, unmasked regions were automatically detected areas. Some of these areas were manually removed from the ROI (Region of Interest) Manager because they were clearly identified as not the void regions, e.g., areas with highly disproportionate dimensions representing fragments of reinforcing fibers, evident phase inclusions, cracks near voids, etc. The calculations were performed for the remaining areas identified as voids. The identified voids had micrometric sizes, ranging from a dozen to several hundred micrometers. All samples’ mean calculated void ratio was e = 0.0257 (2.57%).

**Figure 7:**
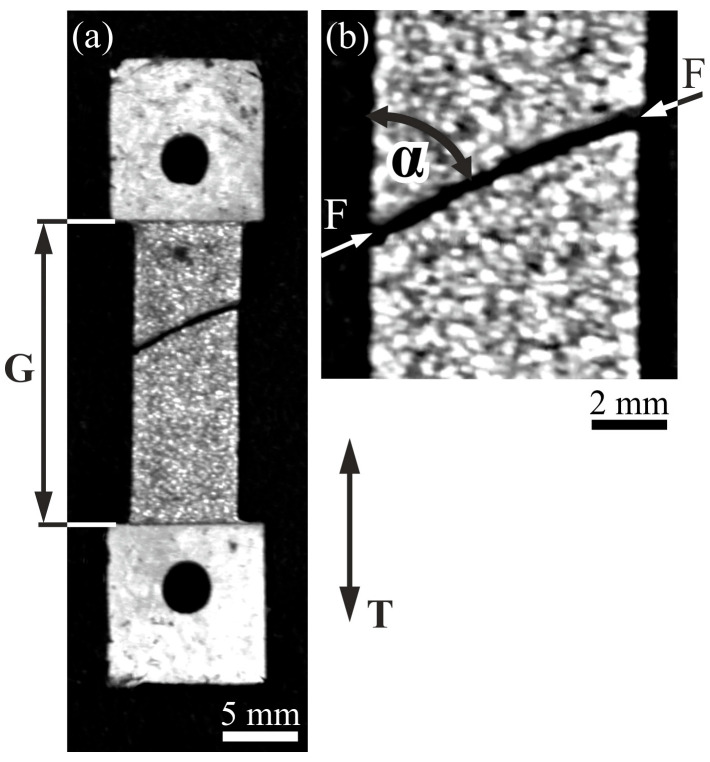
(**a**) The shape of the specimen for tension tests and (**b**) the fracture area. T—tension direction, F—fracture line, G—gauge section, (OLM).

Uniaxial tension tests were performed on the plate-like samples (Figure 7a) until fracture. The samples were cut so that the reinforcement fibers were arranged nearly parallel to the tensile axis T. Figure 7a shows an optical image of the typical fractured sample. The fracture F formed in the gauge section G is visible. As observable in Figure 7b, the fracture is not rectilinear and does not propagate perpendicularly to the edge of the gauge section. The fracture line F is inclined to the tension direction T by the α angle of about 65°.

**Figure 8:**
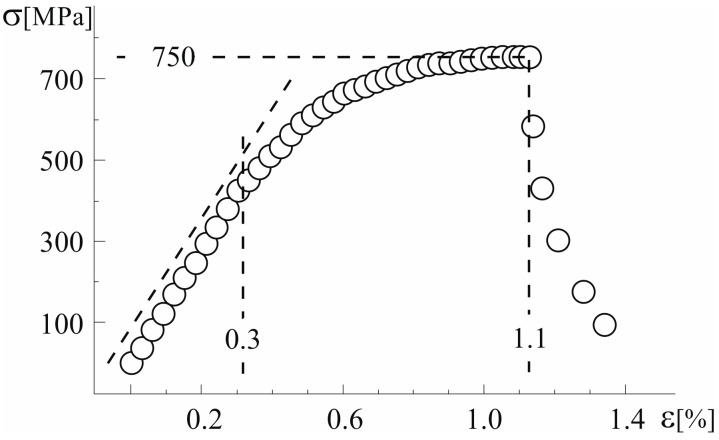
Characteristic stress–strain curve of plate-like samples of the Al_61_Cu_27_Fe_12_ composite.

Figure 8 shows a typical stress-strain curve of the tested plate-like samples of the Al_61_Cu_27_Fe_12_ composite. The linear range in the σ(ε) curve is limited to the strain value of about 0.3%. The tensile test data analysis for each investigated specimen allowed the determination of a breaking stress value of about 750 MPa. The fracture of the samples occurred after reaching a strain of about 1.1%. Standard deviation is provided and limited by the rims of the measurement points.

**Figure 9:**
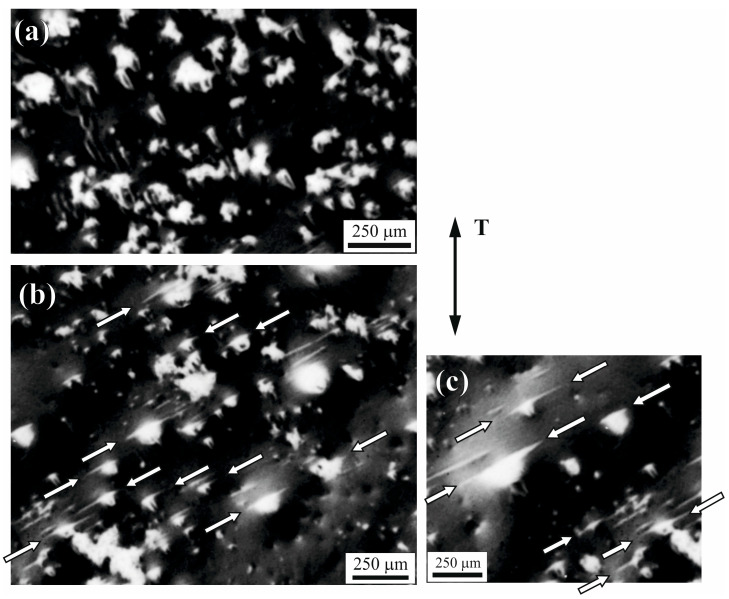
Micrographs of a typical microstructure of parallel slices microsection visualized on the surface of a plate-like sample near the fracture area (**a**) before the tension test, (**b**,**c**) after the tension test, T—tension direction, (SEM, SE mode), the arrows mark the pairs of void streaks.

The microstructure of fractured samples in the gauge section area, especially near the fracture line F (Figure 7b), differs from the microstructure of samples before tensile tests. Figure 9 shows a typical microstructure of parallel slices visualized on the plate-like sample’s gauge section before (Figure 9a) and after (Figure 9b,c) tensile test. The bright regions visible on the micrographs represent voids. The voids appear as bright regions due to their shape and surface orientation. The void’s surfaces, specifically angled to the main observation surface, allow for easier escape of secondary electrons, and the incidence angle of the void’s surface leads to an increased secondary electron yield, which gives the brighter image in void regions. The voids existing in samples before the tensile tests had irregular shapes and were randomly distributed in the sample volume. Analysis of the microstructure of fractured samples after the tension tests shows that in many of the previously visible bright regions, the pairs of streaks on opposite sides were created (marked by arrows in Figure 9b,c). The streaks are located mainly along straight lines, parallel to each other. The directions of these lines correspond approximately to the direction of fracture line F. Some kind of alignment that can be slightly visible in a small area in Figure 6a is not related to voids, but rather to the composite’s phase structure, as the elongation direction is similar to the W withdrawal direction, which also corresponds to the direction of the reinforcing fibers. The elongated regions mentioned above have a different shade and shape from the discussed bright elongation of void streaks and do not occur as pairs.

**Figure 10:**
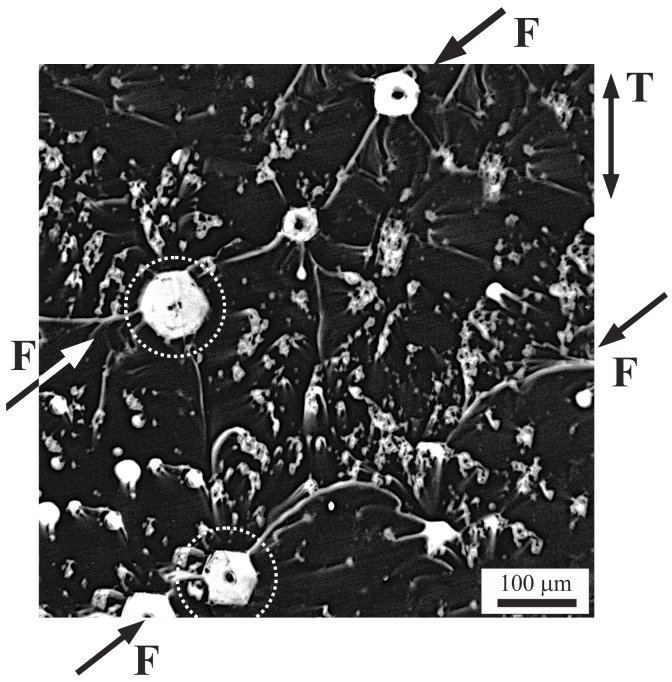
Micrograph of a typical microstructure of parallel slices microsection visualized on the surface of a plate-like sample near the fracture area after chemical etching, T—tension direction, (OLM), F—fracture line marked by arrows.

Afterwards, the surface of the fractured samples was subjected to chemical etching to examine the streaks that appeared more thoroughly. As a result, the bright regions with streaks have been more clearly visible (Figure 10). The micrographs’ analysis allows us to state that some of the streaks are evidently connected, combining such defects. Also in this case, the connecting direction corresponds approximately to the direction of the fracture line F (Figure 7b). Chemical etching revealed the characteristic shapes of some voids marked, for example, by dotted white rims in Figure 10. The cross-sections of such empty spaces, whose plane contained the tension direction T, were often hexagonal. This shape could have been formed under the influence of the β-phase with the six-fold crystallographic symmetry, which is the dominant phase and surrounds the voids [21]. In the case of the voids with hexagonal cross-sections, microcracks appear at the corners of the hexagons, often at opposite corners of the fracture line F.

**Figure 11:**
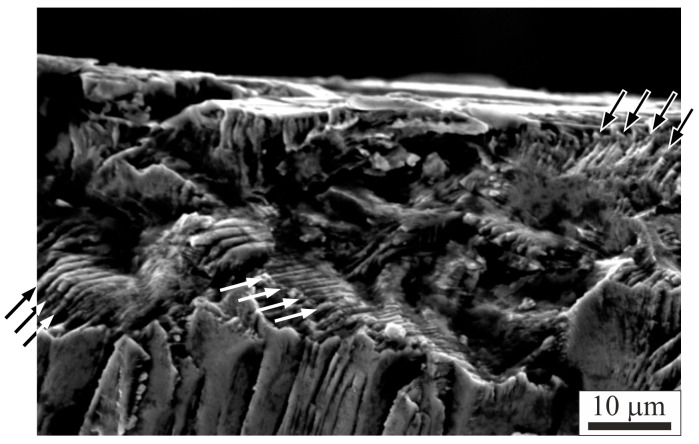
Micrograph of a typical fracture surface of a composite plate-like sample, (SEM). Different arrow colors were used to improve the clarity of the figure.

The typical fracture surface of a plate-like sample is presented in Figure 11. The tensile fracture surface reveals a well-formed stepped pattern, marked in example by arrows in Figure 11. The stepped pattern on the fracture surface was visible in the composite matrix, approximately parallel to the fiber direction.

**Figure 12:**
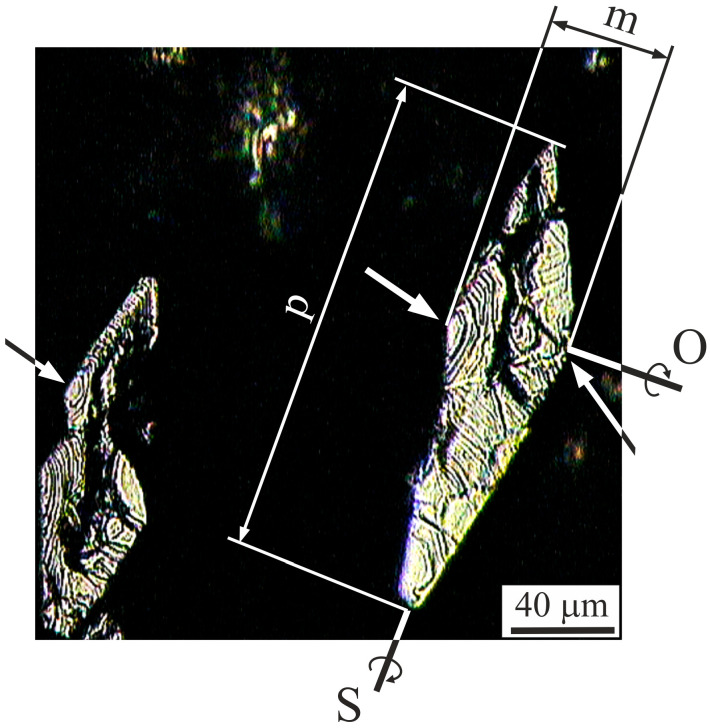
Micrograph of a typical fracture surface of a reinforcement fiber, (OLM). O, S—axes of rotation, m and p—fiber dimension lines.

The stepped pattern in the form of stair steps is also visible on the fracture surface of reinforcement fibers. Figure 12 shows the exemplary cross-section of reinforcement fibers in the fracture area. The stair steps (marked by the arrows) are visible on the fibers’ sections, and the steps are arranged along the semicircles centered at the rhombuses’ corners at a higher angle. There are no observed fractures along the interface boundaries of the reinforcement and the matrix of the composites.

The analysis and comparison of the composites’ microstructure before and after simple uniaxial tensile tests allows one to deduce that the cracks initiate at voids and progress mainly through the voids in the composite matrix and across the reinforcement fibers. The streaks observed with the voids after tensile tests represent the micro-cracks of the voids which appear on their surface when stress increases. The micro-cracks are created in a direction inclined at about 65° to the direction of the tension force. The reinforcement fractured across the fibers in a direction comparable to fracturing in the matrix. The fibers cracked on the stair steps, and there was no delamination at the reinforcement–matrix boundary.

Additionally, it was found that the tensile force direction initiates with the evolution of the voids’ surface (visible in micrographs as changes in the voids’ shape), and formation of micro-cracks. The direction of the micro-cracks on the voids affects the direction of the fracture. The occurrence of the stair-steps morphology may suggest some features of brittle fracture. However, the fracture surface is not perpendicular to the direction of tensile force, and no V-shaped stair step cracks are observed, typical for brittle fracture.

The quasicrystalline i-phase that occurs at the reinforcement–matrix interface (Figure 5) provides a strong interface boundary. The load is effectively transferred from the matrix to the reinforcement. The composite areas where the voids occurred become the regions with the lowest durability of composites and most vulnerable to fracturing. Hence, the cracking process starts at the voids.

As can be seen from the microstructure observations, a slight deviation of reinforcement fibers from the tension direction T exists. The deviation may result in the so-called “bending load” of the fibers. The rhombic shape of the fibers’ cross-section, especially a high difference between their extreme dimensions marked in Figure 12 as *m* and *p* dimension lines, significantly affects the cracking process. Such fibers can be statistically bent more easily relative to the S–axis than the O–axis, which is schematically presented in Figure 12.

## 4. Conclusions

The Al_61_Cu_27_Fe_12_ alloy was directionally solidified by the Bridgman method to obtain novel single-crystalline in situ composites containing quasicrystal phase fraction bonds the matrix and reinforcing fibers. The composites were preliminarily analyzed for the occurrence of voids created during production and their role in the cracking of this new type of composite. The following conclusions were drawn from the analysis of the research results.

The possibility of obtaining novel composites with fibrous reinforcement bonded to a single crystalline matrix by a quasicrystalline phase using directional in situ crystallization during a single solidification process was affirmed.

A new type of composite obtained using the Bridgman method is characterized by a relatively high void content with a ratio of approximately 2.6%. The voids are created throughout composite production, mainly in a single-crystalline matrix.

For the simple uniaxial tension test, the composite’s cracking occurs mainly along the voids and not at the fiber–reinforcement interface, as indicated by the fractures visible in the voids, oriented in the composite cracking direction. Cracks are initiated at voids and progress both through the voids and stair steps in the matrix and the reinforcing fibers. The voids alignment in the matrix affects the composites’ fracturing direction.

Cracking does not occur along the fiber–reinforcement interface as typically for fibrous composites due to the strong bond between them resulting from reinforcement and matrix being obtained in situ in the same process, and the quasicrystalline phase occurs in the interface region.

## Figures and Tables

**Table 1:** Chemical composition in selected points of SEM-EDS microanalysis (Figure 4a).

Point →	1	2	3	4	5	6	7	8
**Al** [at.%]	69.93	70.11	70.58	85.12	85.31	85.07	55.16	56.24
**Cu** [at.%]	20.19	20.27	19.78	0.14	0.17	0.11	44.27	42.57
**Fe** [at.%]	9.88	9.62	9.64	14.74	14.52	14.82	0.57	1.19

**Table 2:** Chemical composition in selected points of SEM-EDS microanalysis (Figure 4b).

Point →	1	2	3	4	5	6	7	8	9
**Al** [at.%]	70.84	69.79	70.05	84.93	85.01	84.94	55.69	56.13	58.14
**Cu** [at.%]	20.07	19.95	19.98	0.15	0.08	0.19	43.53	42.88	40.59
**Fe** [at.%]	9.09	10.26	9.97	14.92	14.91	14.87	0.78	0.99	1.27

## Data Availability

The original contributions presented in this study are included in the article. Further inquiries can be directed to the corresponding author.

## Abbreviations

The following abbreviations are used in this manuscript:

XRD	X-Ray Diffraction
OLM	Optical-Light Microscopy
SEM	Scanning Electron Microscopy
BSE	BackScattered Electrons
SE	Secondary Electrons
EDXS	Energy Dispersive X-ray Spectroscopy
